# Recursive SVM feature selection and sample classification for mass-spectrometry and microarray data

**DOI:** 10.1186/1471-2105-7-197

**Published:** 2006-04-10

**Authors:** Xuegong Zhang, Xin Lu, Qian Shi, Xiu-qin Xu, Hon-chiu E Leung, Lyndsay N Harris, James D Iglehart, Alexander Miron, Jun S Liu, Wing H Wong

**Affiliations:** 1 Bioinformatics Div, TNLIST and Dept of Automation. Tsinghua University, Beijing, 100084, China; 2 Dept of Biostatistics, Harvard School of Public Health, 655 Huntington Ave., Boston, MA 02115, USA; 3 Department of Cancer Biology, Dana-Farber Cancer Institute, Boston, MA, 02115, USA; 4 Medical Proteomics and Bioanalysis Section, Genome Institute of Singapore, Singapore; 5 Dept of Statistics, Harvard University, 1 Oxford St., Cambridge, MA 02138, USA; 6 Department of Statistics, Stanford University, Stanford, CA 94305, USA

## Abstract

**Background:**

Like microarray-based investigations, high-throughput proteomics techniques require machine learning algorithms to identify biomarkers that are informative for biological classification problems. Feature selection and classification algorithms need to be robust to noise and outliers in the data.

**Results:**

We developed a recursive support vector machine (R-SVM) algorithm to select important genes/biomarkers for the classification of noisy data. We compared its performance to a similar, state-of-the-art method (SVM recursive feature elimination or SVM-RFE), paying special attention to the ability of recovering the true informative genes/biomarkers and the robustness to outliers in the data. Simulation experiments show that a 5 %-~20 % improvement over SVM-RFE can be achieved regard to these properties. The SVM-based methods are also compared with a conventional univariate method and their respective strengths and weaknesses are discussed. R-SVM was applied to two sets of SELDI-TOF-MS proteomics data, one from a human breast cancer study and the other from a study on rat liver cirrhosis. Important biomarkers found by the algorithm were validated by follow-up biological experiments.

**Conclusion:**

The proposed R-SVM method is suitable for analyzing noisy high-throughput proteomics and microarray data and it outperforms SVM-RFE in the robustness to noise and in the ability to recover informative features. The multivariate SVM-based method outperforms the univariate method in the classification performance, but univariate methods can reveal more of the differentially expressed features especially when there are correlations between the features.

## Background

Accurate classification of patients with complex diseases such as cancer is crucial for successful treatment of the diseases. High-throughput proteomics techniques based on mass spectrometry (MS) have made it possible to investigate proteins over a wide range of molecular weights in a style similar to gene expression studies with DNA microarrays. The advancement of these techniques has generated many analytic challenges, among which a central task is to discover 'signature' protein profiles specific to each pathologic state (e.g. normal vs. cancer) or differential profiles between experimental conditions (e.g drug responses) from high-dimensional data [1]. The technique of Surface Enhanced

Laser Desorption/Ionization Time-of-Flight Mass Spectrometry (SELDI-TOF-MS) [2] has been used in many recent disease studies [3-5]. Although it is a convenient method for screening a cohort of samples and finding promising protein markers from serum or plasma samples, it suffers from a relatively low sensitivity and specificity and a high noise level [6].

Like in many other biological studies, a key difficulty in such high-throughput studies is the noisy nature of the data, which can be caused by the intrinsic complexity of the biological problems, as well as experimental and technical imperfections. Another difficulty arises from the high dimensionality of the data. Similar to the situation in microarray studies, typically one proteomics investigation only involves several tens of samples but the measured points on the mass spectrum can be in the thousands or more. Even after pre-processing steps such as peak and/or biomarker detection, the dimensionality is usually still larger than or comparable to the sample size. This makes many standard pattern classification algorithms fail. For those that do work theoretically, there is a high risk of overfitting due to the small sample size. Thus, there is an algorithmic need for feature selection in addition to the biological need of discovering a manageable set of key molecular factors (genes or biomarkers) behind the disease. As observed in [7] and other investigations, for machine learning methods such as support-vector machines (SVMs) that can work at high-dimensionality, dimension reduction could still improve the performance dramatically. However, it should be noted that when validating the performance of a classification algorithm with feature-selection steps, the feature selection procedure should also be validated simultaneously to avoid bias in the assessment. Also, due to the small sample size, the cross-validation prediction of the algorithm's performance tends to have a high variance. Thus, we should pay more attention to properties related to generalization ability rather than the prediction performance *per se*. We suspect that failing to do so may be a reason why good results published from one investigation may not be reproduced by other investigations.

Guyon et. al. [7] proposed a SVM-RFE (support vector machine recursive feature elimination) algorithm to recursively classify the samples with SVM and select genes according to their weights in the SVM classifiers. We proposed a method R-SVM with a similar recursive strategy but used a different criterion to evaluate and select the most important genes [8], and a correct scheme to estimate the prediction performance. In this paper, we describe the R-SVM method with a voting scheme for feature selection and compare its performance with SVM-RFE on simulation data and two SELDI-TOF-MS datasets, one on rat liver cirrhosis and another on human breast cancer. We found that cross-validation prediction performances of R-SVM and SVM-RFE were nearly the same, but R-SVM was more robust to noise and outliers in discovering informative genes and therefore has better accuracy on independent test data.

R-SVM and SVM-RFE represent typical machine-learning-based multivariate approaches for the task. Conventional univariate methods are also frequently used for feature selection and classification. We compared the SVM-based methods with the weighted-voting (WV) method [9] on simulation studies, and discussed their respective strengths and weaknesses. Although our real applications were conducted on MS data, the method can be applied broadly to microarray data and other high-throughput genomics and proteomics data.

## Results and discussion

### Simulated data sets

We generated three types of simulated data to investigate the characteristics of the feature selection and classification methods. The basic simulation model for the first two types of data is as follows: Each sample contains simulated expression values of 1000 genes. Among all the genes, 300 are "informative" ones, each following independently the Gaussian distribution *N*(0.25, 1) for class 1 and *N*(-0.25, 1) for class 2. The rest 700 "uninformative" genes follow independently *N*(0,1) for both classes. For each simulation experiment, we generated a training set of 100 samples (50 for each class) and an independent test set of 1000 samples (500 for each class). The two types of simulated data were generated by adding noises to this general model in different ways. The first type (Data-G) mimics the situation where some of the gene expressions in some samples are outliers. For the informative genes, we randomly chose 5% of expression values in all samples as outliers by making them to follow *N*(0.25, 100) for the class-1 sample and *N*(-0.25, 100) for the class-2 sample. The second type of simulated datasets (Data-S) is constructed to contain 5% "outlier samples," which were made by randomly picking 5% of the samples and increasing the standard deviation of every gene in these samples by 10 fold. We did 100 simulations for each type of the data (Data-G and Data-S).

The above two simulation models are over-simplified in many aspects. To mimic more realistic situations, we generated the third type of simulated data based on a real human breast cancer microarray dataset obtained with Affymetrix U133 Plus 2.0 microarrays. The dataset originally contains 78 estrogen receptor positive (ER+) cases and 54 estrogen receptor negative (ER-) cases. The data were normalized by the GCRMA algorithm [10,11], and the gene (probe-set) expression levels were log2-transformed. According to our previous experiments as well as published work (e.g., [12,13]), the ER status is one of the most predominant partitioning factors for molecular classification of breast cancer. We therefore took the differentially expressed genes between the two classes as "truly informative" genes. We extracted the genes whose average differences between the two classes are greater than 0.3, which gave us 16,722 genes (if we use t-test tofind the differentially expressed genes, there will be 24,414 genes come out with FDR-adjusted p-value cutoff 0.05). From these genes, we randomly selected 300 to be the "informative genes" in the simulation dataset. Then, we randomly took another 700 genes and "force" them to be uninformative by permuting their sample labels. By combining the "informative genes" and "uninformative genes", we obtained a simulated dataset in which there are 300 informative genes and 700 uninformative genes. The correlations among the informative genes and among the uninformative genes are the same as those in the original dataset. From this dataset, we randomly took 45 ER+ and 30 ER- samples as the training set, and used the remaining samples as independent test set. We generated 100 sets of data by this strategy. We call this type of datasets *Data-R *in our experiments.

### Real SELDI proteomics data sets

We applied the two SVM-based methods to two real SELDI-TOF-MS proteomics datasets. The first dataset is from a rat model used to discover serum biomarkers of liver cirrhosis [14]. It contains serum protein profiles of 26 normal rats and 69 thioacetamide-induced liver cirrhosis rats. The biomarker function of Ciphergen ProteinChip software 3.0 detected 93 biomarkers from the raw data. They were normalized according to their mean values, small values were truncated to 1 and all biomarkers were log transformed. The second proteomics dataset came from a human breast cancer study. We obtained plasma samples from 75 breast cancer patients and 61 healthy women [15]. The plasma samples were pH-fractionated and analyzed by SELDI-TOF-MS. The Ciphergen ProteinChip software 3.1 detected 98 biomarkers, which were preprocessed in the same way as for the rat liver cirrhosis data.

### Comparison of R-SVM with SVM-RFE on simulated datasets

We first compared the performance of R-SVM to SVM-RFE on Data-G and Data-S. For each of these datasets, we applied R-SVM and SVM-RFE to perform gene selection, built SVM models on training data with selected genes, and tested the models on the independent test data. Experiments were done 100 times for each data type. The following aspects of performances are inspected at each level of gene selection: number of SVs (support vectors, see Method) used in the SVM model, test error, the percentage of true informative features recovered in the selection, and for Data-S the number of outlier samples used as SVs. Tables 1 and 2 show the relative improvements of R-SVM over SVM-RFE with regard to these factors averaged on the 100 experiments for each data type, as well as the p-values (by paired t-test) of the differences. The cross-validation (CV) errors on training sets are similar between the two methods so the comparison is not shown in the tables (Slight improvement of R-SVM over SVM-RFE can be observed on average errors but the improvement is not significant).

It can be seen on both Data-G and Data-S that at most of the selection levels, especially at those lower levels (with fewer selected genes), the number of SVs used by R-SVM is 5 %~8 % fewer than that of SVM-RFE, indicating the better generalization ability of R-SVM. One can also see that at the same selection level, R-SVM recovers significantly more of the informative genes than SVM-RFE, and the improvement is about 3 %~7 % at lower selection levels. These two factors can explain the observation that independent test errors of R-SVM were significantly lower than that of SVM-RFE (by 5–10%) at lower selection levels. On the Data-S with outlier samples, R-SVM also shows a better ability to avoid taking the outlier samples as SVs (R-SVM reduces up to 94% of the outlier SVs than SVM-RFE).

As simulation models for Data-G and Data-S are too simplistic, we compared on Data-R and the results are shown in Table 3. It can be seen that the improvement of R-SVM over SVM-RFE with regard to both the number of SVs used and the number of informative genes recovered are more significant. In terms of testing errors on the independent data, SVM-RFE outperformed R-SVM initially at high selection levels. When fewer genes were selected, R-SVM gradually out-raced SVM-RFE, and the improvement of R-SVM over SVM-RFE became more obvious as the number of selected features decreased. This tendency was also observed on Data-G and Data-S. It is likely due to the fact that R-SVM selected more informative features and that it is more important to include truly informative ones when fewer features are used in a classifier.

### Application on the rat liver cirrhosis data

We applied R-SVM and SVM-RFE to the two real SELDI-TOF-MS datasets. Table 4 shows the results on the rat liver cirrhosis data. Since there is no independent test set and there is no standard answer about the true informative features, we only list the cross-validation errors and the number of SVs in Table 4. It can be seen that at some selection levels R-SVM achieved smaller CV errors, while at others SVM-RFE gave smaller CV errors, but the differences are not significant. However, at most levels, R-SVM uses fewer SVs than SVM-RFE. (Note that in Tables 4 and 6, the number of SVs is the average among the cross-validation experiments, different from that in Tables 1 and 2.)

The top 6 biomarkers selected by R-SVM were reported for further biological investigation. They are listed in Table 5 along with their t-statistics and ROC statistics. We see that the top 6 markers are all significantly correlated with the sample classification on their own, but not necessarily be ranked at the top according to the univariate criterion. Among the top 6 biomarkers, the 3,495 Da protein was down-regulated in the liver cirrhosis rats. On-chip purification and tryptic digestion was conducted. Combined data from PMF (peptide mass fingerprint) and MALDI-TOF/TOF MS/MS spectra suggested that this 3495 Da protein shares homology to a histidine-rich glycoprotein [14]. It has been reported that the mRNA of this gene was found to be specifically expressed only in liver [16]. We speculate that down-regulation of histidine-rich glycoprotein in cirrhotic liver may be a manifestation of loss of normal liver function, including secretary pathways upon treatment with thioacetamide [14].

### Application on the human breast cancer data

The results of R-SVM and SVM-RFE on the breast cancer dataset are listed in Table 6. Error rates and SV numbers on this dataset were higher than in the rat liver cirrhosis study, due to the complexity of the problem under study and individual variations among human individuals. Still, we found that R-SVM tends to use fewer SVs at most selection levels and, hence, may have a better generalizability than SVM-RFE.

The minimal CV error rate (17.6%) is achieved at the 8-feature level by R-SVM. These 8 markers and their t-statistics and ROC statistics are listed in Table 7. Six of the top 8 R-SVM selected markers are called significant by t-statistics (p-value<0.01; 5 of them with p-value<0.0001). The top marker, Marker-5, has an AUC (area under the ROC curve) of only 0.775, but the 8 markers jointly classify 82.4% of all cases correctly. The AUC of the SVM model built on the R-SVM-selected with 8 markers is 0.928, much larger than that of the best single marker, Marker-5. Using direct on-chip sequencing provided by Ciphergen, one important peptide (Peptide A) was identified and was selected for further study. Follow-up biological study showed that this peptide may be an important indicator of the disease status of breast cancer patients [15].

We also applied the Random Forest (RF) method [17] to this human breast cancer dataset. With the DecreaseGiniDistance criterion [17], we selected the 8 most important markers from this data, of which 6 are also among the 8 markers selected by R-SVM. The out-of-bag error reported by the RF method was 25.7%, which is higher than the minimal CV error of 17.6% reached by R-SVM. However, these two error rates are not directly comparable. The out-of-bag error reported by RF is based on a resampling approach, which makes the effective sample size of its training set smaller than the full size and, therefore, causes the estimated prediction error to be biased upwards. When the sample size is small, the bias of resampling error without any correction can be large. On the other hand, although cross validation error by the CV2 scheme (see Method) is unbiased, selecting the minimal error among multiple levels of feature eliminations may lead to a slightly over-optimistic estimate. Taking these two factors together, we conclude that the two tested methods performed similarly on this dataset.

### Comparison with the univariate method

Many researchers believe that it is beneficial to use multivariate methods to analyze microarray and proteomics data as genes usually work in collaborative ways rather than as independent factors. However, univariate methods are still useful for identifying differentially expression features and continue to play important roles in many applications. Therefore it is worthwhile to compare the performances of the SVM-based methods with more conventional univariate approaches, of which we take the weighted-voting (WV) method as a representative in this work.

Tables 8 and 9 show the comparison of R-SVM and WV on Data-G and Data-S. It can be seen that R-SVM outperformed WV on Data-G in both test accuracy and the recovery of informative genes at all selection levels, and the improvement is very significant. However, R-SVM was significantly inferior to WV in both aspects on Data-S, and the difference was more obvious when fewer genes are selected. These observations suggest that R-SVM is more robust than WV to outlier values spreading randomly in the dataset, but suffers more when some samples are entirely corrupted. Indeed, although R-SVM uses the class means to represent the samples for feature selection, the standard SVM uses only boundary samples (SVs) in building the classifiers, thus a few outlier samples can make big effect on degrading its performance. (On this specific simulation model, the effect of the outlier samples can be easily cancelled by taking a normalization step on the samples.)

Table 10 gives the comparison of R-SVM and WV on Data-R. It is interesting to note that, unlike on Data-G or Data-S, R-SVM is superior to WV in terms of test accuracy on independent test sets, but is inferior to WV in terms of the number of recovered informative genes. The differences become smaller when fewer genes were used, but they are still significant. This phenomenon has not been observed in the other types of simulated data, and is likely caused by the fact that some genes in Data-R are correlated as in real microarray and MS data, whereas the genes in Data-G and Data-S are independently generated. As a multivariate method, SVM uses the genes not according to their individual differences between the classes, but rather according to the collaborative information of multiple genes. Thus, some correlated genes will not be selected due to the redundancy of information, even if they are all differentially expressed. This is not a disadvantage for SVM when the major goal is better classification. However, if the goal includes discovering *all *genes that are informative, new strategies will be needed for improving SVM-like methods on this aspect.

We should note that all our simulation designs (Data-G, Data-S, and Data-R) are favorable to univariate methods since the true underlying classification information is in the differential expression of individual genes. The models haven't consider any collaborative effects (not even additive effects), thus they bias in favor of the single-variable approach *a priori*. We expect that in more complicated situations when combinatorial effects play major roles, SVM-based methods will perform even better.

## Conclusion

High-throughput genomics and proteomics data open a new route to the classification of complex diseases. Machine learning methods for feature selection and classification have been playing active roles in analyzing such data. We compared two similar methods, SVM-RFE and R-SVM, both adopting recursive procedures to select features according to their importance in SVM classifiers. The major difference between the two methods is the criteria used for evaluating the contribution of genes. Although the two methods did not differ significantly in their cross-validation performances, it appeared that R-SVM is more robust to severe noise and outliers and can recover more informative genes. The successful application of R-SVM on the rat liver cirrhosis SELDI data and human breast cancer SELDI data show that the proposed strategy can help to identify biologically important markers.

We compared R-SVM with a representative univariate method, the weighted-voting method on simulated data. Although the comparison is limited in scope (especially, no combinatorial effects have been simulated in the models), some interesting insights regarding their respective strengths and weaknesses are observed. The SVM-based method performs better in terms of the classification accuracy, but univariate methods can reveal more of the differentially expressed individual features. A more systematic comparative study of the univariate and multivariate methods can be helpful for better understanding the nature of the methods and problems.

## Availability and requirements

AR code package of the R-SVM method and a Linux-based executable package are freely available.

Project name: R-SVM (R and Linux versions)

Project home page: 

Operating systems: R version: Windows XP, Linux; Linux version: Linux.

Programming languages: R, C/C++

Other requirements: R for the R version; SVMTorch (provided) for the Linux version

License: free

## Methods

### Assessing the performance of feature selection

Since an independent test set is not available in many investigations, cross-validation (e.g., leave-one-out cross-validation or LOOCV) is often used to assess the accuracy of classifiers. It should be noted that feature selection results may vary with even a single-case difference in the training set when the sample size is small. In some literature, feature selection steps were external to the cross validation procedures, i.e., the feature selection was done with all the samples and the cross-validation was only done for the classification procedure. We call this kind of cross validation CV1, with examples including [7,9,18-21]. As pointed out by [22-24], CV1 may severely bias the evaluation in favor of the studied method due to "information leak" in the feature selection step. For example, in a pilot study, we generated 100 samples with 1000 features for each sample, all coming invariably from the Gaussian distribution *N*(0,1). We randomly assigned the samples into two classes ("fake-classes"). Since the data set is totally non-informative, the faithful CV error should be around 50% no matter what method is used. But by CV1 scheme we could achieve a CV error as low as 0.025 after recursive feature selection, which shows that the bias caused by the improper cross-validation scheme can be surprisingly large. A more proper approach is to include the feature selection procedure in the cross validation, i.e., to leave the test sample(s) out from the training set before undergoing any feature selection. In this way, not only the classification algorithm, but also the feature selection method is validated. We call this scheme CV2 and use it in all of our investigations throughout. For the above "fake-class" data, the error rate evaluated by CV2 was always around 50% regardless of the specific method used for feature selection and classification.

### The relative importance of features in SVM classifiers

As a powerful and popular multivariate machine-learning method, SVMs have been widely used in biological classification problems. The key idea of the SVM [25-27] is to maximize the margin separating the two classes while minimizing the total classification errors. The theory and algorithm of SVM has been described in many papers and books (e.g., [26-29]), and several sets of SVM codes are publicly available such as the SVMTorch [30] we use. The SVM can be extended to its nonlinear forms by using proper kernels to replace the inner-products. However, information is usually far from sufficient for reliably estimating nonlinear relations for microarray or MS data. We therefore use the linear SVM here and take it as a reasonable first-order approximation to the "truth" with our limited data. The decision function of a linear SVM is:



where **x **is the gene/protein expression vector of a sample, **x**_*i *_is that of sample *i *in the training set (*i *= 1,2,...,*n*), *y*_*i *_∈{+1,-1} is its corresponding class label,  is the vector of weights of the features, and *b *is a scalar offset. The *a*_*i *_'s and *b *are estimated from the training set. Only those samples closest to the separating boundary (called support vectors or SVs) have non-zero *a*_*i *_'s, and therefore the function *f*(**x**) is a linear combination of only the SVs. For a new sample **x**, the sign of the decision function *f*(**x**) predicts the class it belongs to, and the absolute value of *f*(**x**) represents how far the sample is from the boundary. Theoretical investigations show that the proportion of SVs in the training set reflects an upper bound of the expected generalization error (error rate of predictions on future samples) [28].

Our goal is to select a subset of features with the maximum discriminatory power between the two classes. Since the feature dimension is large and the sample size is small, there are usually many combinations of features that can give zero error on the training data. Therefore, the "minimal error" criterion cannot work. Intuitively, it is desirable to find a set of features that give the maximal separation between the two classes of samples. Taking the possible unbalanced sample size into consideration, we define the following measure:



where *n*_1 _and *n*_2 _are the numbers of samples in class 1 and 2. The larger *S *is, the better separated the two classes are. Considering (1) and denoting the means of feature *j *in the two classes as  and , we get:



where *d *is the total number of features, and *w*_*j *_is the *j*th component of the weight vector **w **. *S *is equivalent to the cosine of the angle between the normal vector of the separation plane (hyperplane) *f*(**x**) = 0 and the vector **m**^+^-**m**^- ^connecting the two class-means. It is the sum of terms defined on the single features, so we can define the contribution of feature *j *in *S *as



We call *s*_*j *_the contribution factor of feature *j*. It is not only decided by the weight *w*_*j *_in the classifier function, but also decided by the data (the class-means).

An alternative way for measuring the importance of the features is using the square of weight  as derived in SVM-RFE by sensitivity analysis [7]. This can be viewed as using the weighted sums of support vectors in each class as the representatives, i.e.,



The separation between the two representatives can be written as



which can be decomposed into the sum the contributions of every features:



We note that, in the case where distributions of the two classes are both high-dimensional Gaussian with identical covariance matrix ∑, the optimal **w **is Fisher's linear discriminant function of the form **w **= (**m**^+ ^- **m**^-^)^*T*^∑^-1^. Thus, (4) corresponds to the individual component of (**m**^+ ^- **m**^-^)^*T*^∑^-1^(**m**^+ ^-**m**^-^), whereas (7) corresponds to the individual component of (**m**^+ ^- **m**^-^)^*T*^∑^-2^(**m**^+ ^- **m**^-^). It thus appears that (7) may have put too much emphasis on the covariance matrix implicitly estimated from the observed high dimensional data. In contrast, our scheme of using class-means as representatives ties in well with the classical linear discriminant analysis. Using class-means as representatives of the two classes makes the method less sensitive to noise and possible outliers, comparing to using only the few support vectors [31]. A more robust representative for a class of samples is the class-medians (or medoids in higher dimensions), as studied in [32]. However, in datasets we have tested, we did not observe significant differences between using medians and means. This is probably because the distributions of genes are reasonably symmetric so that the medians are close to the means.

### Recursive classification and feature selection

To select a subset of features that contribute the most in the classification, we rank all the features according to *s*_*j *_defined in (4) and choose the top ones from the list. We use this strategy recursively in the following procedures:

Step 0. Define a decreasing series of feature numbers *d*_*0*_*> d*_*1*_*> d*_*2 *_> ... > *d*_*k *_to be selected in the series of selection steps. Set *i *= 0 and *d_0 _= d(i.e.*, start with all features).

Step 1. At step *i*, build the SVM decision function with current *d_*i *_*features.

Step 2. Rank the features according to their contribution factors *s_j _*in the trained SVM and select the top *d*_*i*+1 _features (eliminate the bottom *d*_*i *_– *d*_*i*+1 _features).

Step 3. Set *i *= *i*+1. Repeat from Step 1 until *i *= *k*.

This is an implementation of the backward feature elimination scheme described in pattern recognition textbooks (e.g., [33]) with criteria defined on SVM models at each feature-selection level. It should be noted that this scheme is suboptimal as it does not exhaustively search in the space of all possible combinations. Our choices of the number of iterations and the number of features to be selected in each iteration are very *ad hoc*. Although different settings of these parameters may affect the results, we have observed that, for most cases when the two classes can be reasonably separated with the expression data, the classification performances achieved with different settings were very close to each other, and the majority of features ranked at the top positions were also very stable.

We follow the CV2 scheme to estimate the error rate at each level. In cross-validation experiments, different training subsets generate different lists of features (although many or most of them overlap in usual experiments). A frequency-based selection method is adopted to decide the lists of features to be reported [34]. That is, after the recursive feature selection steps on each subset, we count at each of the *d*_*i *_levels the frequency of the features being selected among all rounds of cross-validation experiments. The top *d*_*i *_most frequently selected features are reported as the final *d_*i *_*features (called the top features).

**Figure 1:**
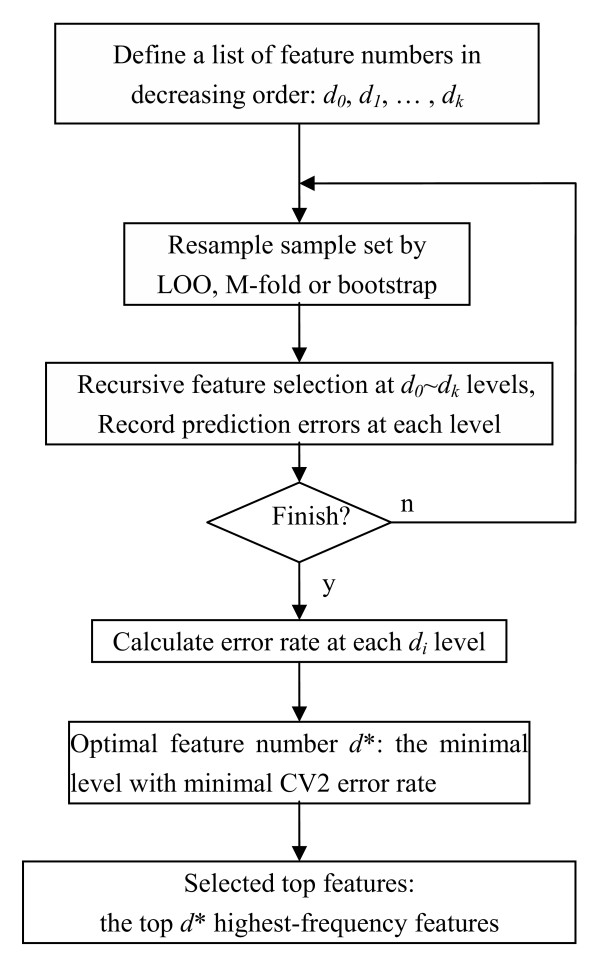
Workflow of the R-SVM algorithm.

In most situations, CV2 errors usually follow a U-shaped curve along the selection steps (feature numbers). Finding the minimal number of features that can give the minimal CV2 error rate is often desirable for real applications. Another realistic consideration is the limited ability of follow-up biological investigations on the selected features. As a compromise, we decide the final number of features to be reported in an experiment by considering both the error rates and the limitation of follow-up biological investigations. For example, in our proteomics applications, we chose to report the number of features that is less than 10 and gives the minimum CV2 error rate for less than 10 features. The entire workflow is depicted in Figure 1; we call this whole scheme R-SVM (recursive SVM).

### The SVM-RFE method

We noted that the original SVM-RFE [7] ranked the genes only once using all samples, and used the top ranked genes in the succeeding cross-validation for the classifier. This is a typical CV 1 scheme which will generate a biased estimation of errors. In order to compare SVM-RFE with the proposed R-SVM algorithm fairly, we wrote our own version of SVM-RFE following the same workflow of R-SVM with the correct cross-validation scheme and the frequency-based selection method, and using the criterion  instead of *s*_*j *_to sort genes.

### The weighted-voting method

The basic idea of the weighted voting method by Golub et al [9] is closely related to the two-sample t-statistics. First, one defines the "correlation" between the expression values of a gene *g *to the classes

*c*_*g *_= [*μ*_1_(*g*) - *μ*_2_(*g*)]/[σ_1_(*g*) + σ_2_(*g*)], (8)

where [*μ*_1_(*g*), *σ*_1_(*g*)] and [*μ*_2_(*g*), σ_2_(*g*)] denote the means and standard deviations of the expression levels of the gene for the samples in class 1 and class 2, respectively. The larger the absolute value |*c*_*g*_| is, the more important the gene is for predicting the class memberships. The genes are ranked by their |*c*_*g*_|'s and the top ones are selected. The class predictor is trained on the set of training samples with the selected genes. For predicting the membership of a new sample ***x ***with expression *x*_*g *_of gene *g*, the vote of gene *g *is *v*_*g *_= *c*_*g *_(*x*_*g *_- *b*_*g*_) where *b*_*g *_= [*μ*_1_(*g*) + *μ*_2_(*g*)]/2. A positive vote means a vote for class 1 and a negative vote is for class 2. The sign of the total vote  is used as the final predictor.

## Authors' contributions

XZ and WHW initiated this project. XZ invented the basic strategy of R-SVM with the help of WHW and they did the study on the two schemes of cross-validation. XZ developed the initial version of the algorithm. XL added the voting scheme, wrote the codes implementing the current algorithm, and did the simulation experiments and most of the computation on the proteomics data. XL and JSL derived the theoretical relationship between R-SVM and SVM-RFE. JSL and WHW guided XZ and XL on analyzing the experiments, comparing the methods, and improving the algorithms. QS, LNH, JDI and AM produced the breast cancer proteomics data and worked on the biological analysis of the results. XX and HEL produced the rat liver cirrhosis data and did the biological analysis and follow-up validation of the results. XZ, XL and JSL executed the writing with input from all authors.

## Figures and Tables

**Table 1:** Comparison of R-SVM and SVM-RFE on Data-G (with gene outliers)

**Level^a^**	**ReduceSV^b^**	**P(sv-diff)^c^**	**ReduceTest^d^**	**P(test-diff)^e^**	**ImproveRec^f^**	**P(rec-diff)^g^**
800	4.01%	1.81E-42	-7.70%	4.72E-03	-3.90%	1.71E-39
600	5.77%	1.74E-49	-2.50%	4.64E-01	-1.70%	5.21E-15
500	6.83%	2.75E-51	-4.00%	1.62E-01	-0.30%	0.079189
400	8.35%	3. 26E-60	2.80%	3.48E-01	1.10%	4.48E-06
300	9.33%	3.83E-58	7.40%	3.65E-02	3.70%	1.77E-31
200	8.22%	1.28E-48	19.20%	6.36E-09	6.30%	5.79E-44
150	8.55%	1.51E-53	19.50%	1.16E-08	7.10%	9.76E-46
100	4.97%	6.20E-22	11.90%	1.83E-04	6.00%	6.43E-40
90	5.84%	1.66E-27	13.70%	4.20E-06	4.60%	1.07E-30
80	5.17%	8.20E-29	12.40%	4.14E-06	4.50%	7.12E-29
70	4.14%	1.46E-27	8.50%	4.77E-04	3.80%	1.05E-24
60	3.10%	1.23E-20	10.20%	3.14E-05	3.40%	4.99E-24
50	2.27%	2.01E-15	10.20%	4.11E-06	2.90%	2.37E-21

^a ^Level: The number of features selected in each recursive step. With all of the 1000 features, there is no difference between R-SVM and SVM-RFE because no feature selection happened.

^b ^ReduceSV: Relative reduction in the mean number of support vectors used by R-SVM comparing to that by SVM-RFE, calculated as: (average #SV_SVM-RFE _- average #SV_R-SVM_)/(average #sv_SVM-RFE_).

^c ^P(sv-diff): The p-value of the observed difference in numbers of SVs, by paired t-test.

^d ^ReduceTest: Relative reduction in the mean test error rates of SVM models with R-SVM-selected features comparing to that with SVM-RFE selected features, calculated as: (average TestError_SVM-RFE _- average TestError_R-SVM_)/(average TestError_SVM-RFE_).

^e ^P(test-diff): The p-value of the observed difference in test error rates, by paired t-test.

^f ^ImproveRec: Relative improvement in the proportion of recovered informative genes by R-SVM than that by SVM-RFE, calculated as: (average #REC_R-SVM _- average #REC_SVM-RFE_)/(average #REC_SVM-RFE_), where #REC is the number of recovered true informative genes with the method stated in the subscript.

^g ^P(rec-diff): The p-value of the observed difference in proportion of recovered true informative genes, by paired t-test.

**Table 2:** Comparison of R-SVM and SVM-RFE on Data-S (with sample outliers)

**Level^a^**	**ReduceSV^b^**	**P(sv-diff)^c^**	**ReduceTest^d^**	**P(test-diff)^e^**	**ImproveRec^f^**	**P(rec-diff)^g^**	**ReduceOSV^h^**	**P(osv-diff)^i^**
800	3.25%	4.49E-41	-65.19%	5.65E-36	-10.14%	3.36E-75	50.37%	5.97E-35
600	5.80%	1.90E-57	-70.27%	3.04E-35	-7.14%	5.18E-56	72.28%	1.10E-49
500	7.02%	8.20E-63	-59.63%	1.81E-37	-5.13%	3.37E-39	80.54%	1.17E-56
400	8.26%	1.68E-67	-41.43%	8.31E-25	-2.57%	4.53E-12	89.04%	2.51E-64
300	7.72%	1.20E-58	-19.14%	2.18E-13	0.75%	4.92E-02	93.44%	7.46E-65
200	7.21%	4.54E-51	-6.53%	2.56E-04	4.00%	7.15E-16	93.91%	1.47E-61
150	9.13%	1.29E-71	2.63%	1.20E-01	6.47%	8.41E-23	93.59%	6.27E-61
100	8.30%	1.42E-64	5.56%	8.04E-04	7.69%	3.50E-22	92.44%	1.33E-61
90	8.36%	2.01E-72	4.31%	1.15E-02	6.99%	8.74E-19	91.37%	2.60E-61
80	8.01%	6.63E-71	4.45%	1.99E-02	6.99%	9.33E-18	90.26%	2.65E-60
70	7.17%	1.29E-67	6.59%	3.78E-04	7.52%	2.80E-16	88.56%	7.55E-62
60	6.67%	2.65E-65	6.16%	2.32E-03	7.27%	5.72E-13	86.38%	2.60E-62
50	5.82%	1.08E-58	7.70%	1.34E-04	7.42%	3.71E-12	83.82%	1.23E-61

^a,b,c,d,e,f,g ^same as in Table 1.

^h ^ReduceOS V: Relative reduction in the number of outlier support vectors (the outlier samples being taken as support vectors) in R-SVM comparing to that in SVM-RFE, calculated as: (average #OSV_SVM-RFE _- average #OSV_R-SVM_)/(average #OSV_SVM-RFE_), where #OSV denotes the number of outlier samples being taken as support vectors by the method mentioned in subscript.

^i ^P(osv-diff): The p-value of observed difference in OVS, by paired t-test.

**Table 3:** Comparison of R-SVM and SVM-RFE on Data-R

**Level**^a^	**ReduceSV**^b^	**P(sv-diff)**^c^	**ReduceTest**^d^	**P(test-diff)**^e^	**ImproveRec**^f^	**P(rec-diff)**^g^
800	15.35%	1.24E-53	-3.59%	1.26E-05	-3.60%	1.50E-23
600	18.65%	3.14E-56	-7.06%	4.09E-04	2.69%	2.20E-09
500	19.58%	7.71E-58	-6.46%	1.79E-03	9.18%	1.24E-37
400	21.07%	1.80E-63	-2.74%	3.22E-05	17.32%	4.25E-59
300	22.51%	5.12E-67	-4.64%	1.26E-05	24.14%	5.43E-65
200	22.16%	9.38E-68	-0.93%	1.83E-04	30.64%	2.25E-71
150	21.78%	4.57E-64	-3.44%	8.74E-04	29.14%	5.86E-71
100	21.01%	3.21E-57	0.31%	3.22E-05	29.95%	7.74E-69
90	22.57%	1.88E-60	-2.52%	3.52E-03	27.51%	9.74E-66
80	22.88%	1.67E-65	1.84%	7.85E-05	27.92%	4.03E-62
70	21.42%	2.96E-59	0.59%	4.09E-04	27.16%	1.15E-58
60	20.20%	1.64E-55	6.16%	1.83E-04	26.83%	2.55E-60
50	18.67%	4.40E-52	4.23%	8.74E-04	25.89%	9.63E-53
40	15.37%	5.66E-46	8.99%	4.69E-06	25.39%	1.09E-55
30	11.85%	6.90E-33	9.61%	1.67E-06	24.19%	2.07E-45
20	7.87%	2.19E-18	11.43%	3.22E-05	20.86%	1.09E-34

^a,b,c,d,e,f,g ^same as in Table 1.

**Table 4:** The CV results on the rat cirrhosis data

**Level**^a^	**R-SVM**		**SVM-RFE**	
	
	CV2^b^	AveSV ^c^	CV2^b^	AveSV ^c^
93	4.2%	14.75	4.2%	14.75
80	4.2%	11.91	4.2%	14.74
70	4.2%	9.95	4.2%	14.73
60	3.2%	9.22	4.2%	13.91
50	3.2%	9.03	4.2%	13.82
40	3.2%	9.02	4.2%	14.65
30	3.2%	8.95	4.2%	13.65
20	3.2%	8.93	4.2%	9.98
18	4.2%	8.14	4.2%	9.97
16	4.2%	8.08	3.2%	7.26
15	4.2%	7.60	3.2%	7.15
14	4.2%	7.54	3.2%	7.94
13	6.3%	7.58	4.2%	7.98
12	6.3%	7.41	4.2%	8.05
11	6.3%	7.65	4.2%	8.02
10	6.3%	7.64	3.2%	9.83
9	5.3%	6.50	3.2%	8.83
8	4.2%	5.97	4.2%	7.01
7	4.2%	6.73	4.2%	6.05
6	4.2%	5.98	3.2%	5.97
5	5.3%	5.94	4.2%	5.05

^a ^Level: The number of features selected in each recursive step.

^b ^CV2: Total cross-validation error rate (CV2 error rate).

^c ^AveSV: Average number of support vectors used in the cross-validations at each level.

**Table 5:** The top 6 R-SVM-selected biomarkers with their t-test and ROC statistics

**m/z (Da)**	**t-test**			**ROC curve**	
	
	**rank**^a^	**t-statistics**	**p-value**	**AUC**	**AUC se**^b^
3526.68	1	11.916	2.05E-19	0.969	0.024
3548.26	3	11.234	4.02E-18	0.955	0.029
1754.12	7	9.784	2.55E-15	0.936	0.034
4195.07	15	5.341	8.46E-07	0.821	0.043
8211.04	30	3.660	4.51E-04	0.712	0.063
4912.63	34	3.339	1.28E-03	0.696	0.057

^a ^Rank by t-statistics

^b ^Standard error of the AUC (area under curve).

**Table 6:** The CV results on the human breast cancer dataset

**Level**^a^	**R-SVM**		**SVM-RFE**	
	
	**CV2**^b^	**MeanSV**^c^	**CV2**^b^	**MeanSV**^c^
98	28.7%	54.65	28.70%	54.65
88	27.9%	50.10	29.40%	55.25
79	29.4%	49.28	30.10%	52.21
71	29.4%	47.48	30.90%	50.88
63	27.9%	44.65	27.90%	48.42
56	27.2%	42.50	27.90%	46.02
50	27.9%	40.04	26.50%	40.13
45	25.7%	38.65	26.50%	40.25
40	24.3%	37.04	27.90%	34.88
36	23.5%	35.16	27.90%	34.51
32	22.1%	33.26	27.90%	30.75
28	22.8%	32.04	27.20%	27.77
25	22.1%	31.24	30.90%	24.61
22	22.1%	31.15	34.60%	23.93
19	22.8%	32.10	30.10%	26.79
17	25.7%	33.26	29.40%	31.28
15	23.5%	35.68	25.70%	35.10
13	19.9%	37.40	26.50%	42.15
11	22.1%	37.83	25.00%	46.03
9	21.3%	42.01	24.30%	50.18
8	17.6%	44.07	22.10%	49.93
7	23.5%	50.29	20.60%	51.43
6	22.1%	54.73	20.60%	52.39
5	22.1%	57.98	20.60%	52.18
4	22.8%	59.75	25.00%	58.92
3	27.2%	78.90	32.40%	77.46

^a,b,c ^Same as in Table 4.

**Table 7:** T-statistics and ROC statistics of the 8 R-SVM-selected markers on the breast cancer data

	**t-test**			**ROC curve**	
**Marker**^a^	**Rank**^b^	**t-statistics**	**P-value**	**AUC**	**AUC se**
^+^Marker-5	1	-5.867	3.31E-08	0.775	0.041
^+^Marker-28	2	-5.229	7.68E-07	0.745	0.043
Marker-29	3	5.169	9.29E-07	0.708	0.044
^+^Marker-58	4	-4.911	2.79E-06	0.754	0.043
Marker-74	6	4.103	7.07E-05	0.700	0.044
Marker-81	10	-2.963	3.61E-03	0.626	0.048
Marker-92	52	1.639	0.104	0.638	0.047
Marker- 97	94	0.162	0.872	0.570	0.049

^a ^The biological study based on these and other data will be published elsewhere. Here we use the relative sequential position of the markers on the m/z axis to represent them.

^b ^Rank ordered by t-statistics among the 98 markers.

^+ ^Biomarkers corresponds to "peptide A"

**Table 8:** The comparison of SVM vs. WV on Data-G

**Level**^a^	**ReduceTest**^d^	**P(test-diff)**^e^	**ImproveRec**^f^	**P(rec-diff)**^g^
800	36.36%	1.16E-17	1.02%	2.13E-06
600	38.95%	6.74E-17	9.49%	2.14E-62
500	39.51%	8.72E-21	14.82%	3.77E-71
400	44.84%	3.86E-23	20.83%	1.68E-79
300	49.75%	6.86E-25	28.72%	3.48E-86
200	54.22%	2.02E-27	36.75%	3.70E-91
150	54.83%	9.37E-30	36.14%	2.65E-86
100	43.56%	6.63E-25	33.61%	4.42E-75
90	42.35%	1.85E-26	31.09%	4.23E-73
80	37.37%	7.35E-25	29.08%	3.79E-67
70	32.23%	1.20E-20	26.54%	9.22E-63
60	27.79%	1.16E-20	24.39%	1.24E-61
50	23.47%	8.64E-15	21.80%	1.83E-53

^a ^Level: The number of features selected in each recursive step.

^d^ReduceTest: Relative reduction in the mean test error rates of SVM comparing to that of WV, calculated as: (average TestError_WV _- average TestError_R-SVM_)/(average TestError_WV_).

^e ^P(test-diff): The p-value of the observed differences in test error rates, by paired t-test.

^f ^ImproveRec: Relative improvement in the proportion of recovered informative genes by R-SVM comparing to that by WV, calculated as: (average #REC_R-SVM _- average #REC_WV_)/(average #REC_WV_), where #REC represents the number of recovered true informative genes with the method stated in the subscript.

^g ^P(rec-diff): The p-value of the observed difference in proportion of recovered informative genes, by paired t-test.

**Table 9:** The comparison of R-SVM vs. WV on Data-S

**Level**^a^	**ReduceTest**^d^	**P(test-diff)**^e^	**ImproveRec**^f^	**P(rec-diff)**^g^
800	-12.32%	1.58E-04	-4.01%	8.77E-33
600	-30.90%	1.38E-19	-0.16%	0.482
500	-40.09%	2.98E-32	-0.03%	0.940
400	-48.92%	2.95E-37	-2.21%	1.29E-11
300	-58.87%	1.54E-44	-6.81%	2.56E-35
200	-64.05%	1.72E-48	-13.73%	2.35E-53
150	-60.96%	1.83E-47	-15.52%	2.15E-52
100	-56.41%	2.62E-49	-19.58%	1.29E-57
90	-52.91%	1.58E-42	-19.14%	1.18E-51
80	-50.73%	2.35E-41	-19.08%	1.31E-51
70	-47.11%	4.02E-40	-18.27%	6.03E-47
60	-43.29%	6.80E-38	-17.58%	1.18E-42
50	-36.01%	1.06E-34	-16.35%	1.34E-37

^a,d,e,f,g ^Same as in Table 8.

**Table 10:** Comparison of R-SVM vs. WV on Data-R

**Level**^a^	**ReduceTest**^d^	**P(test-diff)**^e^	**ImproveRec**^f^	**P(rec-diff)**^g^
800	26.23%	6.15E-11	-14.40%	1.76E-72
600	21.40%	5.51E-08	-20.69%	4.32E-74
500	20.28%	1.12E-09	-22.89%	1.23E-75
400	18.40%	2.70E- 10	-25.16%	2.38E-75
300	14.86%	5.51E-08	-28.52%	1.01E-76
200	18.18%	5.64E-07	-26.47%	3.99E-83
150	13.35%	1.26E-05	-23.87%	4.98E-77
100	13.07%	5.64E-07	-18.37%	1.21E-63
90	13.53%	1.26E-05	-17.91%	1.03E-60
80	15.34%	4.69E-06	-16.69%	1.16E-57
70	12.04%	4.09E-04	-15.49%	4.30E-52
60	12.76%	5.64E-07	-13.95%	4.61E-47
50	9.09%	4.09E-04	-12.68%	3.89E-42
40	8.75%	1.79E-03	-10.50%	1.00E-35
30	8.90%	1.83E-04	-7.77%	2.22E-32

^a,d,e,f,g ^Same as in Table 8.
